# Culture of Kenyan Goat (*Capra hircus*) Undifferentiated Spermatogonia in Feeder-Free Conditions

**DOI:** 10.3389/fvets.2022.894075

**Published:** 2022-07-19

**Authors:** Nakami Wilkister Nabulindo, James Nguhiu-Mwangi, Ambrose Ng'eno Kipyegon, Moses Ogugo, Charity Muteti, Tiambo Christian, Melissa J. Oatley, Jon M. Oatley, Stephen Kemp

**Affiliations:** ^1^Centre for Tropical Livestock Genetics and Health Laboratory, International Livestock Research Institute, Nairobi, Kenya; ^2^Department of Clinical Studies, Faculty of Veterinary Medicine, University of Nairobi, Nairobi, Kenya; ^3^Center for Reproductive Biology, Washington State University, Pullman, WA, United States

**Keywords:** spermatogonial stem cells, goat, culture, markers, serum-free, spermatogonia

## Abstract

The undifferentiated spermatogonial population in mammalian testes contains a spermatogonial stem cell (SSC) population that can regenerate continual spermatogenesis following transplantation. This capacity has the potential to be exploited as a surrogate sires breeding tool to achieve widespread dissemination of desirable genetics in livestock production. Because SSCs are relatively rare in testicular tissue, the ability to expand a population *in vitro* would be advantageous to provide large numbers for transplantation into surrogate recipient males. Here, we evaluated conditions that would support long-term *in-vitro* maintenance of undifferentiated spermatogonia from a goat breed that is endemic to Kenyan livestock production. Single-cell suspensions enriched for undifferentiated spermatogonia from pre-pubertal bucks were seeded on laminin-coated tissue culture plates and maintained in a commercial media based on serum-free composition. The serum-free media was conditioned on goat fetal fibroblasts and supplemented with a growth factor cocktail that included glial cell line-derived neurotrophic factor (GDNF), leukemia inhibitory factor (LIF), stromal cell-derived factor (SDF), and fibroblast growth factor (FGF) before use. Over 45 days, the primary cultures developed a cluster morphology indicative of *in-vitro* grown undifferentiated spermatogonia from other species and expressed the germ cell marker VASA, as well as the previously defined spermatogonial marker such as promyelocytic leukemia zinc finger (PLZF). Taken together, these findings provide a methodology for isolating the SSC containing undifferentiated spermatogonial population from goat testes and long-term maintenance in defined culture conditions.

## Introduction

Spermatozoa are male gametes derived from undifferentiated spermatogonia through a series of cellular divisions (mitotic and meiotic) and morphological transformations collectively referred to as spermatogenesis. The process begins shortly before puberty and is continuous throughout a male's life and this continuity is dependent on the continuous self-renewal of a subset of the undifferentiated spermatogonia population that continues as spermatogonial stem cells (SSCs) (1). The SSCs are unique adult stem cells that contribute genes to subsequent generations making them a perfect target for genetic manipulations and development of transgenic animals through germ cell transplantation of gene-edited SSC (2) and also utilization in the surrogate sire breeding technology (3). In 1994, seminal studies by Brinster and Avarbock (4) demonstrated in mice that SSCs from a donor testis can re-establish spermatogenesis when transplanted to a compatible recipient male (4). This report opened the intriguing possibility of adapting SSC transplantation as a tool for improving reproductive capacity in a variety of animals. Recently, a major step in translating the technique into a utilizable tool for livestock production was achieved by demonstrating that genetically sterile male NANOS2 knockout cattle, pigs, and goats can serve as hosts for donor-derived spermatogenesis following SSC transplantations (3).

Slow rates of genetic gain in low- and middle-income countries (LMICs) represent a major constraint to the improvement of performance in smallholder livestock systems (5) and modeling demonstrates the potential of SSC transplantation technologies to have a dramatic impact (6). Due to their relative rarity in testicular tissue (7, 8), the capacity to isolate and grow SSCs *in vitro* is important to provide sufficient numbers for transplant into a host of surrogate recipient males. For mice, conditions that support long-term maintenance and exponential expansion of SSCs *in vitro* have been devised (9–14). Under supporting conditions, *in-vitro* expanded mouse SSCs can engraft in recipient testes following transplantation and produce colonies of long-lasting donor-derived spermatogenesis. To date, similar accomplishments have not been reported for SSCs of livestock, yet some progress has been made in growing primary cultures with undifferentiated spermatogonial characteristics for cattle and pigs (15–17).

Efficient propagation of SSCs *in vitro* requires recreation of the cognate microenvironment or niche, which the cells reside *in vivo* (13, 14, 18). A series of studies with mouse spermatogonia have demonstrated the importance of feeder cells, growth factor supplements, and oxygen tension to support SSC maintenance and growth *in vitro* (14). In particular, supplementation of serum-free media with recombinant forms of the growth factors such as glial cell line-derived neurotrophic factor (GDNF), fibroblast growth factor 2 (FGF2), Colony stimulating Factor 1 (CSF1), and stromal-derived factor-1 (SDF-1) is of critical importance for the long-term culture of mouse SSCs, as well as bovine spermatogonia (13, 14, 19, 20). Additionally, the use of feeder cell monolayers to culture SSCs is thought to mimic somatic cell structural support of the *in-vivo* environment and provide undefined growth factors that support the survival and proliferation of the cells. From a practical standpoint, the co-culture of bovine undifferentiated spermatogonia with feeder cell monolayers could be problematic. If the cultured cells are intended for transplantation into the testes of recipient bulls, the presence of feeder cells could have a negative impact on colonization efficiency. Since the main application of SSC is the transplantation into surrogate sires for re-establishing spermatogenesis, the use of feeder cells negatively impacts the ability of the cells to colonize the seminiferous tubules basement membrane (15). The use of monolayer of feeder cells presents a variable component that is difficult to standardize across culture platforms for an array of species (15). Thus, feeder-free culture systems are attractive for use in an SSC transplantation breeding concept.

Outside of cattle, reports on long-term *in-vitro* maintenance of undifferentiated spermatogonia from ruminants are limited. In this study, we devised strategies for isolating a cell population enriched for undifferentiated spermatogonia from the testicular tissue of goats indigenous to Kenya and maintaining them as a primary culture.

Goats are considered to be “climate-smart” livestock in this area due to their resilience and thriftiness in the face of climate change (21). The outcomes of the studies described herein represent a significant step in advancing SSC culture and transplantation as a breeding tool to achieve large-scale and widespread dissemination of desirable genetics in sub-Saharan goat production.

## Materials and Methods

Unless otherwise indicated, reagents and chemicals were purchased from Gibco (Grand Island, New York, USA). All the animal procedures were carried out using 10 pre-pubertal male goats of which five were 3 months old and the other five were 5 months old. The procedures adhered strictly to the approved guidelines of the Institutional Animal Care and Use Committee at the International Livestock Research Institute in Kenya (IACUC Ref. No: 2018-15) and the University of Nairobi (Ref.: FVM BAUEC/2019/243). The testicular tissue was obtained following the orchiectomy of the goats. Semi-open orchiectomy of the goats was done following the routine aseptic procedure (22). Scrotal skin incisions were made on the distal one-third of the lateral aspects to expose the testicles within the tunica vaginalis. Each testicle was exposed and the spermatic cord was ligated before removal. After testicular removal, the incision was sprayed with betadine solution and 0.5 ml of tetanus toxoid vaccine (Antivax Limited), anti-inflammatory drug (dexamethasone), and amoxicillin (Betamox®) were administered to prevent post-castration wound infection. The animals were monitored for 24 h for signs of pain. The testicles were sterilized in 70% ethanol and placed in a beaker with Hank's Balanced Salt Solution (HBSS) supplemented with 100 IU ml^−1^ penicillin and 100 μg ml^−1^ streptomycin on ice. The testes were transported on ice to the laboratory within 3 h. In the laboratory, the testis was cleaned and the cell isolation steps were performed in a sterile hood.

### Testicular Cell Isolation

A two-step cell isolation process by Oatley et al. (15) with minor modifications was used to isolate testicular cells from the goat testes. Briefly, testes were washed in HBSS and disentangled gently to expose seminiferous tubules with blunt-edged forceps. About 150–200 mg of tissue was digested in 0.25 mg/ml of collagenase type IV enzyme and 7 mg/ml DNase I in HBSS for 5–7 min in a water bath at 37°C. Elimination of the interstitial cells was done through gravity sedimentation of seminiferous tubules Of (replace with on) ice and discards the supernatant and five times repeated the same process done. The seminiferous tubules were then incubated with 0.25% trypsin/0.04 EDTA and DNase 7 mg/ml in a water bath at 37°C for 30–35 min. The trypsin reaction was terminated by the addition of fetal bovine serum (FBS). The cell suspension was passed through a 40-μm cell strainer, washed in HBSS twice, using centrifugation, and then enrichment for spermatogonia was done.

### Enrichment Procedure for Undifferentiated Spermatogonia

The cell suspension was overlaid on a continuous 30% Percoll gradient and subjected to centrifugation (600 g at 4°C for 8 min) for separation based on cellular density. The cell suspension was seeded on gelatin-coated six well cell culture plates for differential plating (15, 23). The somatic cells attached at the bottom of the plate, while the SSC remained floating in the media were collected. Single (overnight differential plating only) and double enrichment protocols (Percoll density separation and overnight differential plating) were compared to select the best protocol that results in high concentrated SSC in the testicular cell suspension. Cell fractions obtained after enzymatic digestion were divided into three portions to test enrichment for SSC [cell fraction (1) direct seeding without enrichment, (2) enrichment through differential plating only, and (3) double enrichment through Percoll gradient and differential plating]. The double-enriched portion had the highest number of PLZF-positive cells and transparent colonies with fewer somatic cells; therefore, this protocol was used in the study going forward similar to documented studies (15, 23). After overnight incubation for differential plating, the non-adherent cells (spermatogonia-enriched fraction) were collected, washed, and cultured. The cell concentrations and viability obtained from testicular isolation and the enrichment steps were estimated using a hemocytometer and trypan blue exclusion. The adherent cell population on the gelatin-coated plate made up of a heterogeneous population of somatic cells was harvested by trypsinization and cultured on 10% FBS/Dulbecco's Modified Eagle Medium (DMEM). The cells were mitotically inactivated on confluency by treatment with mitomycin C (20 μg/ml) for 3–4 h, followed by three washes in phosphate-buffered saline (PBS) to remove mitomycin (cells were used as feeder cells in a different study) (data not shown).

### Goat Fetal Fibroblasts Cell Line Establishment

Goat fetuses aged 30–45 days of gestation were collected after the slaughter of pregnant dams (*n* = 5). The head, viscera, and gonads were removed. The tissues were minced and digested with an enzyme solution of 0.25% trypsin, 0.5 mM EDTA, and DNase I (1 mg/ml) in PBS to generate a single-cell suspension. The cells were plated in T75 culture flasks in 10% FBS/Dulbecco's Modified Eagle Medium (DMEM). The cells were mitotically inactivated by treatment with mitomycin C (20 μg/ml) for 3–4 h, followed by multiple washing in PBS. These cells were then used for conditioning of serum-free medium that was used for the culture of SSC.

### Feeder-Free, Serum-Free Culture of Undifferentiated Spermatogonia

The spermatogonial-enriched cell fraction was seeded on laminin-coated plates and three different base media were evaluated after 7 days in culture, namely, MEMα, DMEM/F-12, and StemPro™-34 SFM. StemPro™-34 SFM medium had the highest colony formation, increased number of cells, and was chosen as the medium for continued use in the current study (data not shown). The medium was supplemented with human forms of growth factors: human glial cell line-derived neurotrophic factor (hGDNF) (20 ng/ml; R&D Systems), human fibroblast growth factor 2 (hFGF2) (1 μg/ml; BD Biosciences), human leukemia inhibiting factor (hLIF) (10 ng/ml; R&D Systems), and human stromal-derived factor 1 (hSDF-1) (10 ng/ml; PeproTech, Inc.). The cells at a concentration of 0.02 × 10^6^ cells per well in the StemPro™-34 SFM culture medium (Table 1) were seeded on 96-well laminin-coated plates in the incubator with an air atmosphere containing 5% CO_2_ at 37°C and the media changed every other day. The serum-free medium was conditioned by incubating goat fetal fibroblast monolayer of cells overnight and then passing through a 0.2-μm filter Oatley and Oatley (16). The cells were passaged after 7 days. The colony growth was monitored for up to 45 days and images were taken using the ZEISS Axio Vert.A1 inverted microscope.

**Table 1:** Serum-free media components that support long-term maintenance of goat SSC *in vitro*.

**Media**	**Product company and catalog number**	**Required concentration**
MeM α alpha (1 ×) or Stempro™-34 SFM (1X) or DMEM/F12	Gibco; 41061-029 or Gibco: 10639011 or 11320033	1X
Iron saturated transferrin	Sigma T1283	10 mg/ml
Sodium selenite	Sigma S5261	0.003 M
2-mercaptoethanol	Sigma M3148	100 mM
Insulin	Life technologies 12585-014	4 mg/ml
Putrescine hydrochloride	Sigma P5780	16.1 mg/ml
MEM NEAA (100X) solution	Gibco 11140050	100X
MEM vitamins solution	Gibco 11120052	100X
Glutamine	Gibco 25030024	100X
BSA stempro™	Gibco A100081	1X
Stempro® hESC supplement	Gibco A10006-01	1X
Hepes solution	Sigma H0887	10mM
Penicillin-streptomycin	Gibco 15070-063	(5,000 U/ml)

### Immunocytochemical Staining of Cultured Testicular Cell Populations

Testicular isolate and cultured cells adhered on poly-L-lysine-coated slides and SSC was localized using PLZF marker as described (24) with minor modifications. Briefly, the slides were fixed in 4% paraformaldehyde in PBS for 10 min at room temperature, followed by washing three times with 1X PBS/0.1% (v/v) Triton X-100 (PBST) for 5 min. Then, cells were permeabilized by treating with 0.3% (v/v) Triton X-100 for 15 min. Cells were blocked for unspecific binding by incubation in 10% normal goat serum (w/v) in PBST overnight at 4°C. The cells were washed three times with 1X PBS and incubated at 4°C overnight with primary antibodies diluted in 0.5% bovine serum albumin (BSA) and 0.1% Triton X-100 in PBS. Primary antibodies used: Dead box helicase 4 (DDX4)/VASA polyclonal antibody (bs-3597R, Bioss Antibodies, dilution factor 1:200), PLZF antibody (sc-28319, Santa Cruz Biotechnology, dilution factor 1:200), and NANOS2 Antibody (sc-393868, Santa Cruz Biotechnology, dilution factor 1:200). After overnight incubation, the cells were washed in 1X PBS and stained with fluorescent dye-labeled secondary antibodies (dilution factor 1:1,000) in 0.5% BSA/PBST and incubated for 1 h at room temperature in the dark. Subsequently, the cells were washed with PBS and mounted with ProLong Gold Antifade mounting medium with 4',6-diamidino-2-phenylindole (DAPI) for nuclei staining for viewing under fluorescence microscopy (EVOS M5000 Thermo Fisher microscope) and analysis of images performed (Celleste 5.0 software analyzer). The percentage of undifferentiated spermatogonia positive to PLZF marker was estimated by counting the total number of cells positive for PLZF marker in the enriched cell population and the non-enriched in 10 different fields of view for each slide and dividing by the total number of DAPI-stained nuclei in the fields viewed. PLZF was selected as a marker based on previous studies in sheep (25) and goats (26–28).

### Immunohistochemical Analysis of PLZF Expression in the Goat Testis

Pieces of goat testicular tissue were sectioned and fixed in 10% formalin overnight. The tissue was then dehydrated, embedded in paraffin, sectioned at a thickness of 7 μm, and then adhered to glass slides. The sections were deparaffinized in xylene and rehydrated with a descending series of gradient ethanol and water incubations. Antigen retrieval was done by boiling the slides in sodium citrate buffer (pH 6.0) for 20 min in a water bath at 95°C. Non-specific binding sites were blocked through overnight incubation in 10% normal goat serum/1% BSA in 0.2% Triton X-100 in PBS at 4°C. On the next day, slides were washed in PBS and incubated overnight with the primary antibody at 4°C [1:100 DDX/VASA polyclonal antibody (bs-3597R) and 1:100 PLZF antibody (sc-28319)]. The following day, sections are washed in PBS and then incubated with the secondary antibody for 2 h at room temperature (dilution factor 1:500). Afterward, the tissue was washed in PBS and then a glass coverslip was mounted on the tissue section using the aqueous DAPI-containing medium. Slides were observed under fluorescent microscopy.

### Quantitative PCR Analysis of Expression Levels of Spermatogonial Stem Cell Markers

Quantitative PCR (qPCR) was performed as described previously (29). Total RNA was extracted from SSC colonies on the 30th day of culture using the RNeasy Mini Kit (Qiagen, California, USA) and TRIzol Reagent (Ambion by Life technologies). Then, complementary DNA (cDNA) synthesis was done using the SuperScript III Kit (Invitrogen). The cDNA was quantified and purity was determined by Nanodrop. Quantitative PCR was performed to assess the expression of *PLZF*, B-cell CLL/lymphoma 6 member B (*BCL6B*), and Ubiquitin carboxyl-terminal hydrolase isozyme L1 (*UCHL1*) genes in multiparameter-selected SSC and cultured SSC colonies. The glyceraldehyde-3-phosphate dehydrogenase (*GAPDH*) gene was used as an internal reference gene in the qPCR. The primer sequences used in the reactions were documented for goat SSC gene expression (29) (Table 2). Relative gene expression levels were analyzed using QuantStudio 3 and 5 data analysis software version 1.5.2 using GAPDH served as the internal reference gene. Gene expression data were normalized against GAPDH expression.

**Table 2:** Primer sequences for genes expressed by SSC as described by (29).

	**Gene**	**Annealing temperature**	**Primer sequence**	
1	PLZF	58	GCAACAGCCAGCACTATACTC	Forward
			TACAGCAGGTCATCCAGGTC	Reverse
2	BCL6B	58	GCCACCACCTTTAATTTCTCAC	Forward
			GAAATCAGGCTTCCAGTCTC	Reverse
3	UCHL1	58	GATAAAGCACTTACCCTCAACC	Forward
			GCCTTAACTTACAGACACAAACC	Reverse
4	ID4	56	TGTCACTGAGTTTCATGTCTG	Forward
			AGAAAGTGTTCATTGCCAAGAG	Reverse
5	THY1	56	CTGACCCGTGATACAAAGAAGTG	Forward
			TGAAGTTGGACAGGTAGAGGA	Reverse
6	GAPDH	56	TCAAGAAGGTGGTGAAGCAG	Forward
			CCCAGCATCGAAGGTAGAAG	Reverse

### Statistical Analysis

Mean differences and SEM were determined using the unpaired/two-tailed *t*-test and ANOVA in R software. *P* < 0.05 was considered statistically significant.

## Results

### Isolation of Undifferentiated Spermatogonia From Pre-pubertal Goat Testes

**Figure 1:**
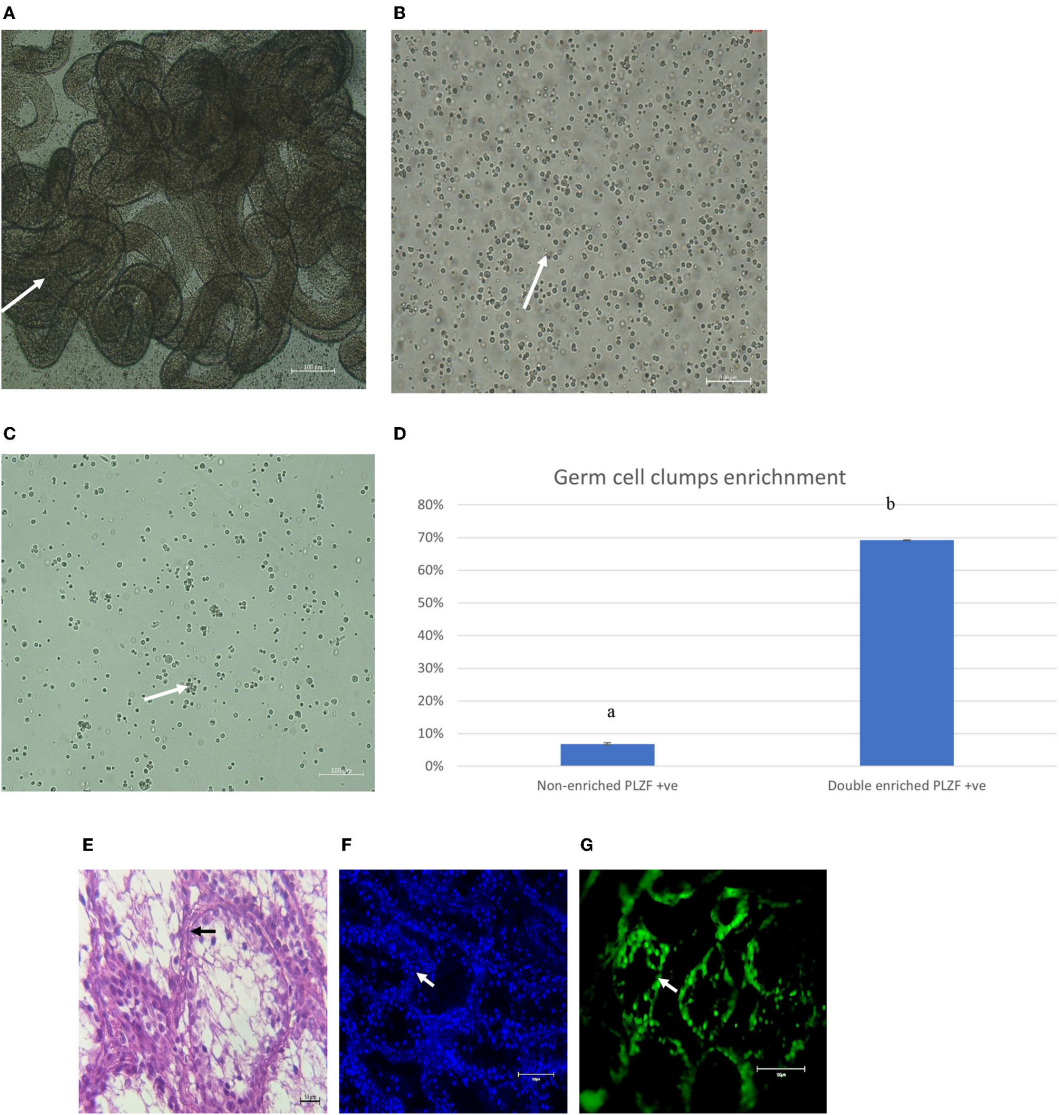
**(A)** Representative images (white arrow) of dispersed seminiferous tubules following initial digestion of the testicular tissue, with interstitial cells between the tubules. **(B)** Unselected single cell suspension following secondary digestion in trypsin. **(C)** Representative image of single cell suspension following multiparameter selection for enrichment of undifferentiated spermatogonia. Magnification factor × 100. **(D)** Percentage of cells in the multiparameter selection population that were determined to express the undifferentiated spermatogonial marker PLZF, different letters a,b represent significant difference in expression of PLZF between non-enriched and double enriched SSC (*P* < 0.05). **(E)** Histological section of pre-pubertal testes stained with Haematoxylin and Eosin (arrows point to spermatogonial within the seminiferous tubules basement membrane). **(F)** DAPI staining of cells in cross-section of the seminiferous tubules (white arrows represent testicular cell nuclei). **(G)** PLZF staining of SSC in cross-section of the seminiferous tubules of pre-pubertal bucks (white arrows point to SSC). (Magnification × 100). Goat testis was used as the positive control.

To obtain starting cell populations for the establishment of primary cultures, we utilized a multiparameter selection process devised previously for bovine testicular tissue (15) but with modifications. Testicular tissue was digested in 0.25 mg/ml collagenase type IV enzyme for 5 min and the obtained seminiferous tubules were digested in 0.25% trypsin/0.04% EDTA trypsin enzyme for 30 min. After initial digestion of the seminiferous tubules, the single-cell suspension was comprised of ~70% undifferentiated spermatogonia, determined based on immunochemical staining for the marker PLZF (Figures 1A–G). Following the enrichment strategy, 2.7–3.2 × 10^6^ (mean ± SEM) cells were isolated per 200 mg of testicular tissue and ~70% of the population was determined to be PLZF + spermatogonia (Figure 1D). Cell viability analysis showed that on average, 77.4% ± 1.2 of the isolated population was live cells.

### Feeder-Free Culture of Multiparameter-Selected Goat Undifferentiated Spermatogonia

**Figure 2:**
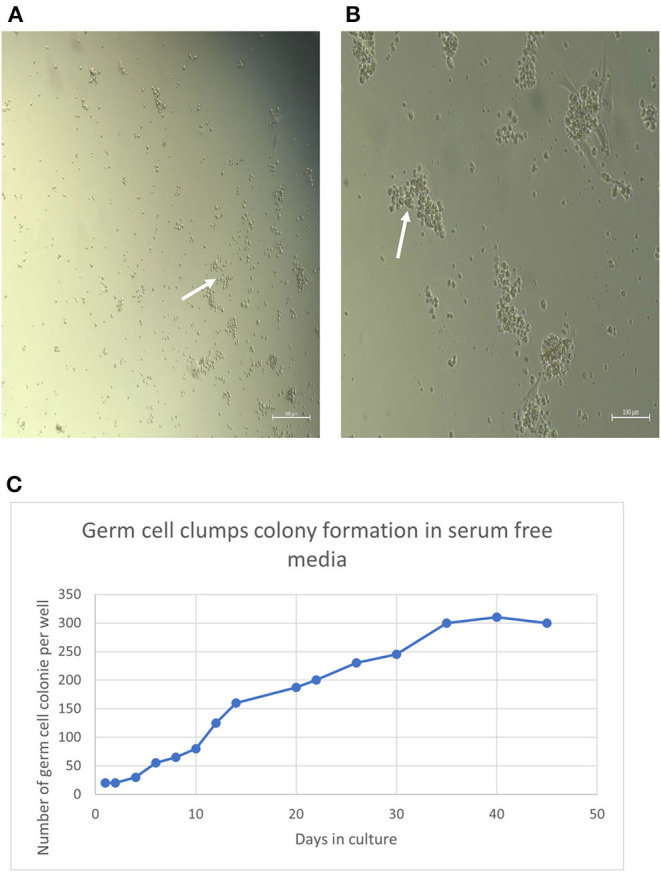
**(A)** Undifferentiated spermatogonia germ cell clumps at day 3 (white arrow) (Magnification factor × 50). Undifferentiated spermatogonia germ cells clumps at day 35 **(B)** (white arrows). Magnification factor × 100. Number of clumps increased concomitant with increasing time of *in vitro* maintenance until day 35 and leveled off thereafter until the end of the analysis period at day 45 **(C)**.

**Figure 3:**
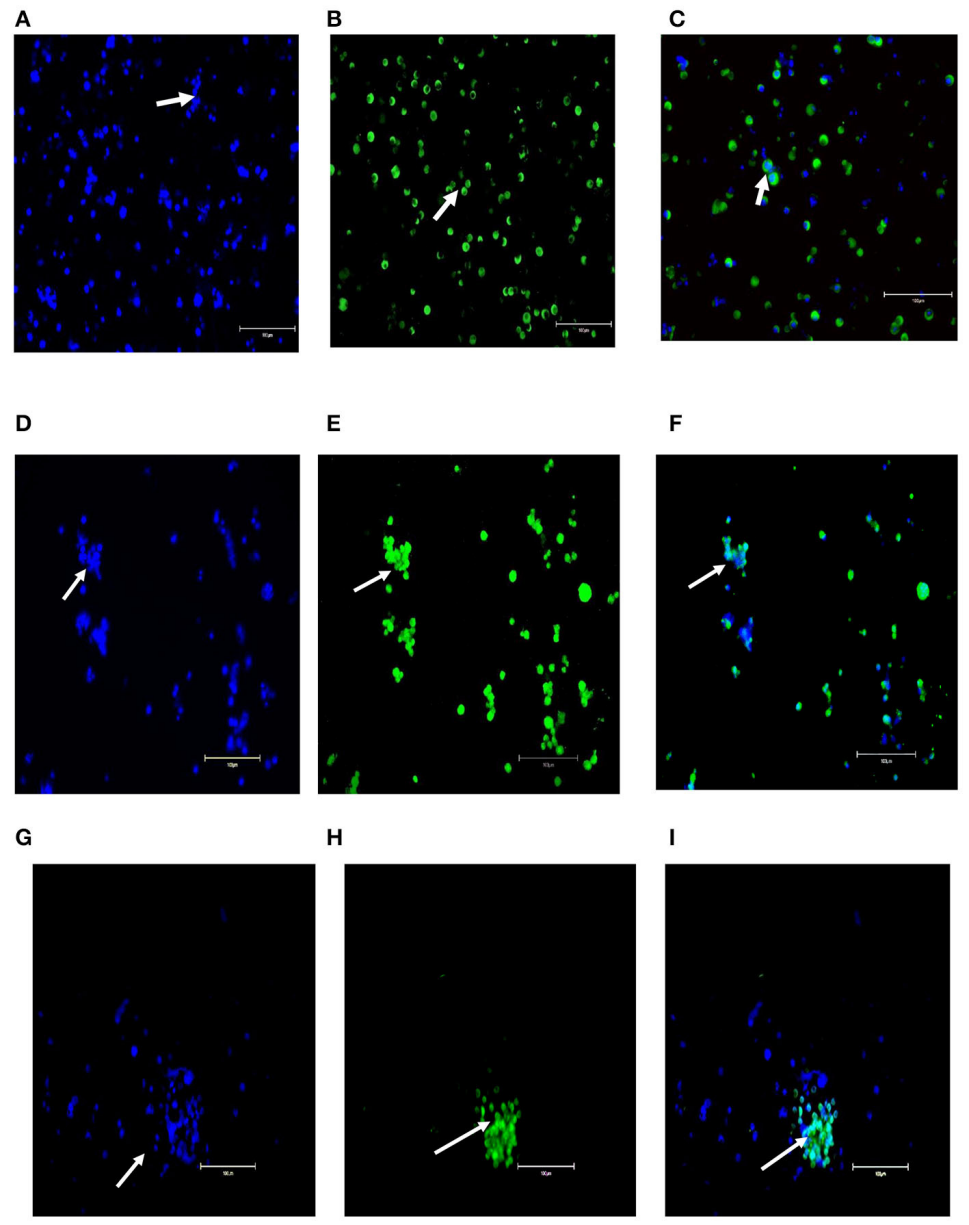
Molecular characterization of primary goat spermatogonial cultures **(A–I)**. **(A)** Staining of the germ cell clumps nuclei by DAPI. **(B)** Staining of germ cell clumps with VASA marker and **(C)** Combined staining of germ cell clumps with DAPI and VASA. **(D)** Staining of the germ cell clumps nuclei by DAPI), **(E)** Staining of germ cell clumps with PLZF marker, and **(F)** Combined staining of germ cell clumps with DAPI and PLZF. **(G)** Staining of the germ cell clumps nuclei by DAPI), **(H)** Staining of germ cell clumps with NANOS2 marker, and **(I)** Combined staining of germ cell clumps with DAPI and NANOS2. Magnification factor × 100. Goat testis was used as the positive control. Arrows point germ cells clumps stained.

**Figure 4:**
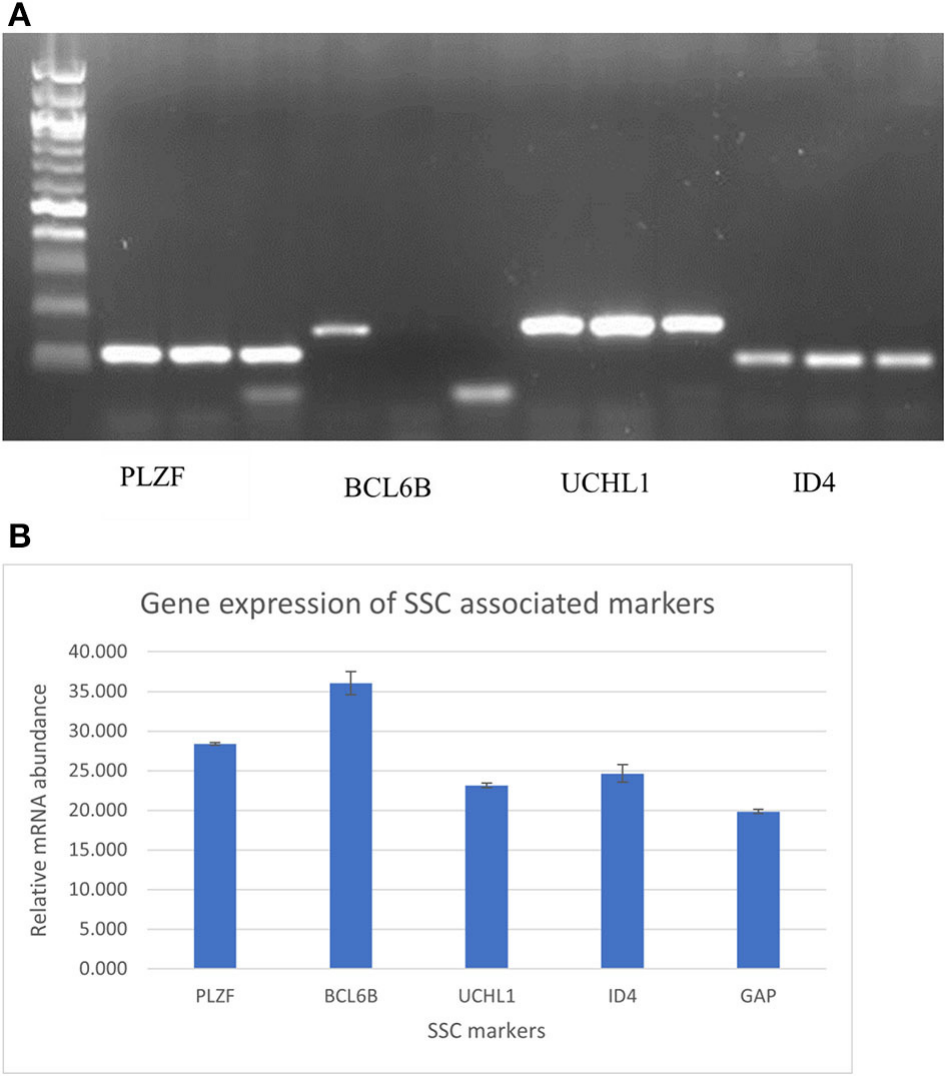
**(A)** PCR and gel electrophoresis analysis for expression of the undifferentiated spermatogonial genes PLZF, BCL6B, UCHL1, ID4 in triplicate. **(B)** RT-PCR analysis for expression of the undifferentiated spermatogonial genes PLZF, BCL6B, UCHL1, ID4 A, and GAPDH. Data are Mean of the triplicate CT values of individual genes ± Standard error of the mean.

After initial plating on laminin-coated plates with serum-free medium, clumps of cells with a germ cell morphology similar to previous reports for other mammalian species appeared at 3 days of culture on average (Figure 2A). The clumps increased concomitant with increasing time of *in-vitro* maintenance until day 35 and leveled off after that until the end of the analysis period at day 45 (Figures 2B,C). To characterize the cultures further, we next used PCR and immunocytochemical staining to assess the expression of molecular markers. Positive immunostaining for the well-defined germ cell markers such as VASA (also known as DDX4), PLZF, and NANOS2 demonstrated that clump-forming cells were goat undifferentiated spermatogonia (Figures 3A–I). In addition, the qPCR analysis revealed the expression of the previously defined spermatogonial markers, including BCL6B, Inhibitor of DNA binding 4 (ID4), and UCHL1 (Figure 4A). Collectively, these findings demonstrate long-term culture and expansion of primary goat undifferentiated spermatogonia.

### Polymerase Chain Reaction Analysis of Spermatogonial Stem Cell Markers Expressed in the Cultured Spermatogonial Stem Cell

Conventional PCR and gel electrophoresis were carried out to ascertain the SSC-related gene expression (PLZF, BCL6B, UCHL1, and ID4) after 1 month of culture of goat germ cell clumps. The results revealed the expression of these genes (Figure 4A). qPCR was also carried out to check for relative quantities of the genes. The ΔCt = ΔCt target–ΔCt internal reference determined relative gene expression levels. GAPDH was used as an internal reference gene in the experiment. All the five genes tested were expressed in the SSC colonies with PLZF and BCL6B having more messenger RNA (mRNA) relative abundance (Figure 4B). Gene expression assessment by PCR analysis for markers of undifferentiated spermatogonia corroborated enrichment *via* the modified multiparameter selection process.

## Discussion

Over the years, livestock genetic improvement has been through a selection of elite sires for breeding. Male germplasm has played a vital role in genetic improvement. For example, over the years, tropical regions have benefited from the importation of top bull semen for artificial insemination in dairy cattle. This is primarily aimed at improving production traits, fertility, longevity, and disease resistance in subsequent generations. It suffices that artificial insemination has revolutionized the dairy industries of developing countries. Kenya is an example where milk production has dramatically increased over the years. Additionally, this has led to the availability of purebred dairy cattle as replacement heifers to apply superior genetics. Comparatively, the small ruminant's production system has lagged in fully exploiting the genetic potential. This is more apparent in indigenous goats with limited application of reproductive technologies. There is potential through reproductive technologies to facilitate the dissemination of superior germplasm. Thus, the opportunity to reap the benefits of genetic gains has not been fully tapped. Transplantation of SSCs from elite bucks into the locally adapted bucks would provide an alternative breeding method for disseminating genetically superior semen within these existing infrastructural limited breeding systems, especially in sub-Saharan Africa.

The exploitation of SSC transplantation in livestock production has been limited by several factors, mainly, the lack of optimized protocols for long-term maintenance of SSCs in culture and the lack of methods to prepare ideal SSC recipients. Recently, there was a published report on the development of ideal surrogates, boars, bulls, and bucks for SSC transplantation (3). These surrogates were genetically deficient in the germ cell layer, but the somatic cell structure was intact and functional. This was achieved by knocking out the *NANOS2* gene, which is responsible for germ cell layer survival and development. These gene-edited surrogate sires exhibited complete donor-derived spermatogenesis with normal semen levels (3). These new developments are a significant milestone toward the utilization of the surrogate sires breeding approach in livestock production systems. With these advancements, there is an increased need for the establishment of a long-term SSC culture system in livestock that will enable amplification of the low numbers of isolated SSC to millions for use in transplantation.

In the last decade, efforts to culture SSCs from livestock species have been made with varying success. Previous reports have reported the isolation and long-term maintenance of goat SSC in culture (26, 29). However, there is a morphological variation of the SSC compared to rodent SSC, whose stem cell capacity has been confirmed through transplantation and donor-derived spermatogenesis (11, 12). Rodents SSC long-term cultures have been established and the conditions and characterization parameters have been optimized. Morphologically, rodent SSC in culture forms germ cell clump colonies with loosely attached cells that resemble many grapes. A previous comparison of the SSC morphology in mice, pigs, and bovine revealed a conserved similarity across the three species (15, 30). Therefore, a similar morphology of SSC forming germ cell clumps would be expected in goat species. In the current study, the culture conditions used supported the formation of germ cell clumps of goat SSC, which were almost identical to SSC clumps reported in rodent cultures. Additionally, the goat SSC germ cell clumps, stably expressed PLZF, a conserved specific SSC marker in rodents and goats. We successfully cultured goat SSC on serum-free medium yielding cultures morphologically similar to those described in rodents and livestock species (15, 30). The media components of the serum-free medium included StemPro™-34 SFM, bovine serum albumin, and StemPro™-34 Nutrient Supplement and were supplemented with the human forms of growth factors such as GDNF, bFGF, LIF, SDF, and other additives. The StemPro™-34 SFM medium has been documented for the successful culture of rodent SSC (10) and sheep SSC (15, 23).

Some previous studies have reported the use of serum for the propagation of goat SSC in culture (26, 31). However, in the current study, preliminary trials using serum in the culture medium did not result in the formation of typical SSC germ cell clumps, but rather formed tightly packed spheres that resembled masses of somatic cells outgrowth (data not shown here). The current study further confirms that the use of serum in SSC culture inhibits self-renewal and causes the proliferation of somatic cells. Therefore, the results in the current study provide further information for the development of long-term maintenance of goat SSC in culture.

Stem cell renewal, survival, and functions within the stem cell niche are dependent on the somatic cell support comprised mostly of Sertoli cells and Leydig cells among others. One way of mimicking this microenvironment is through culturing SSC on the feeder layer. However, in the current study, goat SSC was efficiently cultured on laminin-coated plates (feeder-free) at 37°C in a 5% CO_2_ incubator for extended periods (and formed typical germ cell clumps morphology). Laminin base coating has previously been utilized in the feeder-free culture of SSC and successfully established feeder-free bovine SSC (15). This is the first report of serum-free feeder culture of goat SSC.

In the current study, primary cultures of SSC were obtained by isolating testicular cells from pre-pubertal goats 3–6 months of age; at this age, the majority of gonocytes have transformed into undifferentiated type A spermatogonia. During the pre-pubertal period, the undifferentiated spermatogonia are mitotically active with regenerative capacity, which is a hallmark of SSCs (32, 33). Thus, donor bucks had a maximum concentration of SSC in the testis compared with gonocytes and differentiating spermatogonia. Consequently, the germ cell clumps obtained in the study could have been derived from a population with a high number of undifferentiated spermatogonia and few gonocytes of which both are spermatogonial stem cells with similar biochemical characteristics. This further provides evidence together with morphology and expression of markers that the population of testicular cells isolated and cultured in the current study was made up of undifferentiated spermatogonia.

We believe that this is the first report of serum-free, feeder culture of goat SSCs and culture of SSCs in African livestock breeds in Africa. The conditions established here can be used to develop robust long-term cultures of goat SSCs enabling an improved rate of genetic gain in goats. With the continued food insecurity due to the increase in the human population, efficient and effective breeding technologies are needed to match the demand for animal products. This necessitates the exploitation of advanced technologies such as surrogate sires breeding technology, where SSCs are utilized in transplantation.

This will be highly applicable in arid and semi-arid areas where huge numbers of goats are kept by pastoralist communities and the infrastructure to support other reproductive technologies is not sustainably available. The current technology will be applicable through the initial introduction of surrogate sires as rotational breeding bucks to community goat flocks for the propagation of desirable genetics through natural mating within the existing infrastructure. The male offsprings born from rotational surrogate sire mating will continue the natural propagation of the desirable genetics within the flocks without the need for additional or improved infrastructure. Kenya has research centers such as the International Livestock Research Institute, universities, and other livestock research institutes that have the capacity to produce surrogate sires and distribute them to goat farmers for improved productivity.

## Data Availability Statement

The original contributions presented in the study are included in the article/supplementary material, further inquiries can be directed to the corresponding author/s.

## Ethics Statement

The animal study was reviewed and approved by Institutional Animal Care and Use Committee at International Livestock Research Institute in Kenya (IACUC Ref. No: 2018-15) University of Nairobi (Ref.: FVM BAUEC/2019/243).

## Author Contributions

NN: carried out the research activities and manuscript writing. SK: manuscript review and editing. JO: played a great role in transfer of technology from Washington state university to ILRI, manuscript review and editing. MJO: training of the ILRI team on the isolation and cell culture of undifferentiated spermatogonia. TC: reproductive platform and project supervision. CM: research technician for the cell culture lab activities-asssited in cell culture. MO: research technician for molecular activities-assisted in castartions and PCR works. AK and JN-M: manuscript review. All authors contributed to the article and approved the submitted version.

## Funding

This study was funded by the CGIAR Research Program on Livestock (https://livestock.cgiar.org/) and the Centre for Tropical Livestock Genetics and Health (Grant Number: OPP01127286).

## Conflict of Interest

The authors declare that the research was conducted in the absence of any commercial or financial relationships that could be construed as a potential conflict of interest.

## Publisher's Note

All claims expressed in this article are solely those of the authors and do not necessarily represent those of their affiliated organizations, or those of the publisher, the editors and the reviewers. Any product that may be evaluated in this article, or claim that may be made by its manufacturer, is not guaranteed or endorsed by the publisher.

## References

[1] de Rooij DG. The spermatogonial stem cell niche in mammals. In: Sertoli Cell Biology. 2nd ed. Academic Press (2015). p. 99–121. 10.1016/B978-0-12-417047-6.00004-1

[2] Dobrinski I. Transplantation of germ cells and testis tissue to study mammalian spermatogenesis. Anim Reprod. (2006) 3:135–45.

[3] Ciccarelli M Giassetti MI Miao D Oatley MJ Robbins C Lopez-Biladeau B. Donor-derived spermatogenesis following stem cell transplantation in sterile NANOS2 knockout males. Proc Natl Acad Sci USA. (2020) 117:24195–4195:atl Acad1073/pnas.2010102117 32929012 10.1073/pnas.2010102117 PMC7533891

[4] Brinster RL Avarbock MR. Germline transmission of donor haplotype following spermatogonial transplantation. Proc Natl Acad Sci USA. (1994) 91:11303–13034atl Ac1073/pnas.91.24.11303 7972054 10.1073/pnas.91.24.11303 PMC45219

[5] Marshall K Gibson JP Mwai O Mwacharo JM Haile A Getachew T. Livestock genomics for developing countries—African examples in practice. Front Genet. (2019) 10:1–13. 10.3389/fgene.2019.00297 31105735 PMC6491883

[6] Gottardo P Gorjanc G Battagin M Gaynor RC Jenko J Ros-Freixedes R. A strategy to exploit surrogate sire technology in livestock breeding programs. G3 Genes, Genomes, Genet. (2019) 9:203 Gen 10.1534/g3.118.200890 30563834 PMC6325890

[7] Tegelenbosch RA de Rooij DG Tagelenbosch RAJ de Rooij DG Tegelenbosch RA de Rooij DG. A quantative study of spermatogonial multiplication and stem cell renewal in the C3H/101 F1 hybrid mouse. Mutat Res. (1993) 290:193ouse. 10.1016/0027-5107(93)90159-D 7694110

[8] Oatley JM Brinster RL. The germline stem cell niche unit in mammalian testes. Physiol Rev. (2012) 92:577RevB 10.1152/physrev.00025.2011 22535892 PMC3970841

[9] Kanatsu-Shinohara M Inoue K Ogonuki N Morimoto H Ogura A Shinohara T. Serum- and feeder-free culture of mouse germline stem cells. Biol Reprod. (2011) 84:97prodn 10.1095/biolreprod.110.086462 20844279

[10] Kanatsu-Shinohara M Miki H Inoue K Ogonuki N Toyokuni S Ogura A. Long-term culture of mouse male germline stem cells under serum-or feeder-free conditions1. Biol Reprod. (2005) 72:985rod 10.1095/biolreprod.104.036400 15601913

[11] Kanatsu-Shinohara M Muneto T Lee J Takenaka M Chuma S Nakatsuji N. Long-term culture of male germline stem cells from hamster testes. Biol Reprod. (2008) 78:611rod 10.1095/biolreprod.107.065615 18094355

[12] Kanatsu-Shinohara M Ogonuki N Inoue K Miki H Ogura A Toyokuni S. Long-term proliferation in culture and germline transmission of mouse male germline stem cells. Biol Reprod. (2003) 69:612rod 10.1095/biolreprod.103.017012 12700182

[13] Kubota H Avarbock MR Brinster RL. Growth factors essential for self-renewal and expansion of mouse spermatogonial stem cells. Proc Natl Acad Sci USA. (2004) 101:16489–6489:atl Aca1073/pnas.0407063101 15520394 10.1073/pnas.0407063101 PMC534530

[14] Helsel AR Oatley MJ Oatley JM. Glycolysis-optimized conditions enhance maintenance of regenerative integrity in mouse spermatogonial stem cells during long-term culture. Stem Cell Rep. (2017) 8:1430–43017ell Rep1016/j.stemcr.2017.03.004 28392219 10.1016/j.stemcr.2017.03.004 PMC5425612

[15] Oatley MJ Kaucher AV Yang Q-E Waqas MS Oatley JM. Conditions for long-term culture of cattle undifferentiated spermatogonia. Biol Reprod. (2016) 95:14. 10.1095/biolreprod.116.139832 27251094

[16] Oatley JM Oatley MJ. Feeder-Free Method for Culture of Bovine and Porcine Spermatogonial Stem Cells. Google Patents. Publication Number WO/2013/122864; WO2013122864 (2013).

[17] Suyatno S Kitamura Y Ikeda S Minami N Yamada M Imai H. Long-term culture of undifferentiated spermatogonia isolated from immature and adult bovine testes. Mol Reprod Dev. (2018) 85:236–49. 10.1002/mrd.22958 29480937

[18] Kubota H Avarbock MR Brinster RL. Culture conditions and single growth factors affect fate determination of mouse spermatogonial stem cells. Biol Reprod. (2004) 71:722rodv 10.1095/biolreprod.104.029207 15115718

[19] Yang L Wu W Qi H. Gene expression profiling revealed specific spermatogonial stem cell genes in mouse. Genesis. (2013) 51:83 Wu 10.1002/dvg.22358 23175476

[20] Oatley JM Oatley MJ Avarbock MR Tobias JW Brinster RL. Colony stimulating factor 1 is an extrinsic stimulator of mouse spermatogonial stem cell self-renewal. Development. (2009) 136:1191–1916:pmentO1242/dev.032243 19270176 10.1242/dev.032243 PMC2685936

[21] Bjornlund V Bjornlund H Van Rooyen AF. Why agricultural production in sub-Saharan Africa remains low compared to the rest of the world hya historical perspective. Int J Water Resour Dev. (2020) 36:20ater 10.1080/07900627.2020.1739512

[22] Yami A. Castration of sheep and goats. Technical Bulletin No.18. In: Merkel RC editor. Ethiopia Sheep and Goat productivity Improvement Program. (2009). p. 12.

[23] Binsila BK Selvaraju S Ghosh SK Ramya L Arangasamy A Ranjithkumaran R. EGF, GDNF, and IGF-1 influence the proliferation and stemness of ovine spermatogonial stem cells *in vitro*. J Assist Reprod Genet. (2020) 37:2615–615:0st Repr1007/s10815-020-01912-5 32821972 10.1007/s10815-020-01912-5 PMC7550450

[24] Reding SC Stepnoski AL Cloninger EW Oatley JM. THY1 is a conserved marker of undifferentiated spermatogonia in the pre-pubertal bull testis. Reproduction. (2010) 139:893ionl 10.1530/REP-09-0513 20154176

[25] Binsila KB Selvaraju S Ghosh SK Parthipan S Archana SS Arangasamy A. Isolation and enrichment of putative spermatogonial stem cells from ram (*Ovis aries*) testis. Anim Reprod Sci. (2018) 196:9prod 10.1016/j.anireprosci.2018.04.070 29861343

[26] Pramod RK Mitra A. *In vitro* culture and characterization of spermatogonial stem cells on Sertoli cell feeder layer in goat (*Capra hircus*). J Assist Reprod Genet. (2014) 31:993 1001. 10.1007/s10815-014-0277-1 24958548 PMC4130931

[27] Bahadorani M. Comparative immunohistochemical analysis of VASA, PLZF and THY1 in goats and sheep suggests that these markers are also conserved in these species. J Cytol Histol. (2011) 2:126. 10.4172/2157-7099.1000126

[28] Bahadorani M Hosseini SM Abedi P Hajian M Hosseini SE Vahdati A. Short-term *in-vitro* culture of goat enriched spermatogonial stem cells using different serum concentrations. J Assist Reprod Genet. (2012) 29:39t Re 10.1007/s10815-011-9687-5 22160429 PMC3252412

[29] Sharma A Shah SM Tiwari M Roshan M Singh MK Singla SK. Propagation of goat putative spermatogonial stem cells under growth factors defined serum-free culture conditions. Cytotechnology. (2020) 72:489nolo 10.1007/s10616-020-00386-8 32124159 PMC7225234

[30] Giassetti MI Ciccarelli M Oatley JM. Spermatogonial stem cell transplantation: insights and outlook for domestic animals. Annu Rev Anim Biosci. (2019) 7:385v Ani 10.1146/annurev-animal-020518-115239 30762440

[31] Bahadorani M Hosseini SM Abedi P Abbasi H Nasr-Esfahani MH. Glial cell line-derived neurotrophic factor in combination with insulin-like growth factor 1 and basic fibroblast growth factor promote *in vitro* culture of goat spermatogonial stem cells. Growth Factors. (2015) 33:181acto 10.3109/08977194.2015.1062758 26154310

[32] Curtis SK Amann RP. Testicular development and establishment of spermatogenesis in holstein bulls. J Anim Sci. (1981) 53:1645–645:1 Sci Am2527/jas1982.5361645x 7341622 10.2527/jas1982.5361645x

[33] Murta DVF Costa DS Santos MD Faria FJC. Somatic and germ cell proliferation during post-natal development of the testis in the wild boar (Sus scrofa scrofa). Anim Reprod Sci. (2010) 119:154od 10.1016/j.anireprosci.2009.11.003 19944546

